# Cannabis Use and Age‐Related Changes in Cognitive Function From Early Adulthood to Late Midlife in 5162 Danish Men

**DOI:** 10.1002/brb3.70136

**Published:** 2024-11-07

**Authors:** Kirstine Maarup Høeg, Rasmus Ljungbeck Frodegaard, Marie Grønkjær, Merete Osler, Erik Lykke Mortensen, Trine Flensborg‐Madsen, Gunhild Tidemann Okholm

**Affiliations:** ^1^ Unit of Medical Psychology, Section of Environmental Health, Department of Public Health University of Copenhagen Copenhagen Denmark; ^2^ Center for Clinical Research and Prevention Copenhagen University Hospital – Bispebjerg and Frederiksberg Frederiksberg Denmark; ^3^ Section of Epidemiology, Department of Public Health University of Copenhagen Copenhagen Denmark; ^4^ Center for Healthy Aging University of Copenhagen Copenhagen Denmark

**Keywords:** cannabis, cognitive decline, cognitive function, cohort study

## Abstract

**Introduction:**

Cannabis is by far the most widely used and abused drug listed on the Drug Enforcement Administration's Schedule I, which includes drugs with a high potential for abuse. There is evidence of short‐term negative effects of cannabis use on cognition, but only a limited number of studies have explored the association between cannabis use and age‐related cognitive decline. The aim of the present study was to investigate the relationship between cannabis use and age‐related cognitive decline from early adulthood to late midlife.

**Methods:**

The study population consisted of 5162 men who had participated in Danish follow‐up studies on cognitive aging. These studies included scores on the military intelligence test Børge Prien's Prøve from both the conscription assessment (mean age = 20 years; p1 and p99: 18 and 26 years) and from the follow‐up (mean age = 64 years; p1 and p99: 55 and 72 years) as well as extensive data on lifestyle and health from the follow‐up questionnaires. The association between cannabis use and age‐related cognitive decline was investigated in linear regression models.

**Results:**

Men with a history of cannabis use had less cognitive decline from early adulthood to late midlife compared to men without a history of cannabis use. Among cannabis users, neither age of initiation of cannabis use nor frequent use was significantly associated with a greater age‐related cognitive decline.

**Discussion and Conclusions:**

In a sample of more than 5000 men followed for a mean of 44 years, we found no significant harmful effects of cannabis use on age‐related cognitive decline.

## Graphical abstract

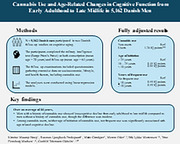

## 1 Introduction

Since the 1960s, cannabis use has been rapidly increasing, especially among younger people, reaching its peak in the late 1970s (World Health Organization nd; Committee on the Health Effects of Marijuana: An Evidence Review and Research Agenda, Board on Population Health and Public Health Practice, Health and Medicine Division, National Academies of Sciences, Engineering, and Medicine 2017). Cannabis is currently classified under Schedule I of drugs by the United States Drug Enforcement Administration, which is a list including drugs with no currently accepted medical use and a high potential for abuse (USC Environmental Health & Safety nd). Recreational use of cannabis is illegal in Denmark; however, according to a recent report from the Danish Health Authority, 9.7% of the Danish population aged 16–44 use cannabis annually (Sundhedsstyrelsen 2022). Given the high prevalence of cannabis use, its potential association with cognitive decline could have significant implications for public health. While there is substantial evidence of the short‐term negative effects of cannabis use on cognition and cognitive development (Dellazizzo et al. 2022; Lubman, Cheetham, and Yücel 2015; Lyketsos et al. 1999; Noorbakhsh et al. 2020), its link to age‐related cognitive decline has been sparsely studied and has shown mixed results (Auer et al. 2016; Lorenzetti, Hoch, and Hall 2020; Lubman, Cheetham, and Yücel 2015; Lyketsos et al. 1999; McKetin et al. 2016; Meier et al. 2012; Watson et al. 2023). For example, in a New Zealand longitudinal study conducted by Meier et al. (2012), frequent cannabis use was associated with steeper cognitive decline over more than 20 years, a decline that was more pronounced the greater the cannabis use (Meier et al. 2012). This study further revealed that adolescent cannabis users were particularly vulnerable to more substantial IQ decline compared to those who initiated cannabis use in adulthood (Meier et al. 2012). Similarly, in an American longitudinal study by Auer et al. (2016), prior cannabis exposure was associated with impaired verbal memory, although it did not appear to affect other cognitive domains (Auer et al. 2016). Contrarily, Lyketsos et al. (1999), in another American longitudinal study, found no significant differences in cognitive decline between heavy cannabis users, light users, and nonusers (Lyketsos et al. 1999). This is consistent with findings from an Australian longitudinal study by McKetin et al. (2016), which suggested that cannabis use was not correlated with accelerated cognitive decline (McKetin et al. 2016). Finally, in an American longitudinal study of older adults with HIV by Watson et al. (2023), occasional cannabis users demonstrated better overall cognitive performance compared to nonusers, but the rates of cognitive decline and everyday function did not vary according to the average cannabis use (Watson et al. 2023).

The present study aimed to investigate the association between cannabis use and age‐related cognitive decline from early adulthood to late midlife in a population of 5162 men. In addition, we aimed to investigate whether this association depended on age of initiation of cannabis use and the number of years of frequent cannabis use.

## 2 Methods

### 2.1 Study Population

This study used data from the Danish Aging and Cognition (DanACo) cohort, which was designed to study predictors of age‐related cognitive decline from young adulthood to late midlife (Grønkjær et al. 2024). The cohort was based on a pooling of two follow‐up studies (LiKO‐15 and DiaKO‐19) with identical design. The studies were based on reassessments of cognitive abilities in late midlife using the conscription board intelligence test (Børge Priens Prøve [BPP]) (Grønkjær et al. 2024), with a mean retest interval of 44 years (p1 and p99: 35 and 53 years). In Denmark, all men must appear before the conscription board between the ages of 18 and 26, though a small proportion is exempt due to certified disqualifying diseases (Forsvarsministeriet 2006). The follow‐up studies compared the military intelligence test results from conscription with those from the follow‐up examination and included a comprehensive questionnaire on socioeconomic, lifestyle, and health‐related factors (Grønkjær et al. 2024). In total, 5340 men participated in the follow‐up studies with an overall participation rate of 14.3% (Grønkjær et al. 2024), but due to technical problems and missing data on cannabis use, 178 participants were excluded, resulting in a study sample comprising 5162 men born in 1949–1961. The examinations at conscription were conducted in 1967–1989, when the men had a mean age of 20 years (Grønkjær et al. 2024) (p1 and p99: 18 and 26 years). The follow‐up examinations were conducted in 2015–2017 and 2019–2022, when the men had a mean age of 64 years (Grønkjær et al. 2024) (p1 and p99: 55 and 72 years).

### 2.2 Cannabis Use

Information regarding former or current use of illicit drugs in Denmark was collected at the follow‐up examination. Information on *cannabis use* was analyzed as a binary variable indicating whether the men had ever used cannabis. Men with a current or former cannabis use at follow‐up will be referred to as cannabis users, while men who have never or almost never used cannabis will be referred to as nonusers. Self‐reported information on *age of initiation* was categorized into three groups: < 18, 18–25, and > 25 years. Information on frequency of cannabis use was only available in LiKO‐15, that included a question on how often (Never/Almost never, Less than once a month, Approximately once a month, A couple of times a month, Approximately once a week, A couple of times a week, Every day/Almost every day) the men had used different types of illicit drugs during different age periods (< 15, 15–18, 19–25, 26–30, 31–40, 41–50, 51–60, > 60 years) as well as within the last 12 months (see Table S1). Frequent cannabis use was operationalized as using cannabis a couple of times a week or more. No frequent use indicated that they had never (i.e., in none of the age periods specified above) used cannabis twice a week or more often, regardless of how long they had been using it. The *years of frequent cannabis use* were calculated by adding the number of years for each age category (e.g., 7 years for the category “19–25 years”) in which the men indicated a frequent use by the definitions specified above, thus assuming a consistent use within each age period. Years of frequent cannabis use included any combination of age periods between initiation and the present, regardless of how recent or how long ago the usage occurred. The years of frequent cannabis use were then categorized into three groups: no frequent use, ≤ 10 years of frequent cannabis use, and > 10 years of frequent cannabis use.

### 2.3 Cognitive Decline

In the present study, *cognitive decline* was defined as the difference in IQ between the two assessments of cognitive ability conducted in early adulthood and late midlife, respectively. Cognitive ability was assessed using BPP, which has been demonstrated to have high reliability and validity and is highly correlated with the full‐scale IQ of the Wechsler Adult Intelligence Scale (Mortensen, Reinisch, and Teasdale 1989; Teasdale et al. 2011). The BPP is a group‐administered and timed intelligence test containing 78 items distributed on four subtests: letter matrices (19 items), verbal analogies (24 items), number series (17 items), and geometric figures (19 items) (Teasdale 2009). The total BPP score corresponding to the number of correct answers to the four subtests with values ranging from 0 to 78 is available from the conscription board and follow‐up examinations. The total BPP scores were linearly converted to a standardized IQ scale with a sample mean of 100 and a standard deviation of 15, using the conscription scores as a reference.

### 2.4 Covariates

*Retest interval* (years) was calculated as the difference between the age at the conscription board and the *age at the follow‐up* examinations. From the follow‐up examinations, both information on self‐reported school education and post‐school vocational training and education was available. Based on this information, a combined measure of *years of education* was calculated. *Years of weekly extreme binge drinking* were calculated using the frequency of consuming ≥ 10 units of alcohol on the same occasion (seven response categories from Never/Almost never to every day/Almost every day) for each age period, as well as the duration of the period, assuming consistent alcohol use within each age period. Information on *smoking* was used to categorize the men into never, former, or current smokers at the time of the follow‐up examination. *Use of other illicit drugs* was a dichotomous variable categorizing the men according to whether they had ever used other illicit drugs than cannabis (e.g., amphetamine, LSD, or heroine).

Psychiatric and somatic morbidity was based on information on hospital admission diagnoses from the Danish Psychiatric Central Research Register (PCRR) and the Danish National Patient Registry (DNPR) (Lynge, Sandegaard, and Rebolj 2011; Mors, Perto, and Mortensen 2011). *Psychiatric morbidity* was categorized as a binary variable based on whether the men had ever been registered with an in‐ or outpatient diagnosis in PCRR before the follow‐up examination. For somatic morbidity, information from DNPR was used to calculate the *Charlson Comorbidity Index score* (CCI) (for more details, see Charlson et al. 1987). Higher CCI indicate more somatic morbidity.

### 2.5 Statistical Methods

The characteristics of the study sample were presented for the full sample and separately for cannabis users and nonusers. Unadjusted differences were analyzed using Welch's *t*‐test. In Table 1, the change in cognitive functions from baseline to follow‐up is referred to as IQ change and is presented with mean and standard deviation.

**TABLE 1:** Characteristics of the study sample of 5162 men in relation to ever cannabis use.

	Have used cannabis			
Variables	No	Yes	Total	*p* value^a^
*n* (%)	3134 (60.7)	2028 (39.3)	5162	
IQ (mean [SD])				
Conscription	99.51 [15.19]	100.77 [14.67]	100.00 [15.00]	0.003
Follow‐up	92.68 [14.95]	95.48 [13.77]	93.78 [14.56]	< 0.001
**IQ change**	**−6.82 [9.54]**	**−5.29 [9.88]**	**−6.22 [9.70]**	**< 0.001**
Age at examination, years (mean [SD])				
Conscription	20.03 [2.08]	20.25 [2.20]	20.12 [2.13]	< 0.001
Follow‐up	64.50 [4.03]	63.54 [4.03]	64.12 [4.06]	< 0.001
Retest interval length	44.47 [4.37]	43.30 [4.15]	44.01 [4.32]	< 0.001
Years of education (mean [SD])	12.49 [4.47]	12.99 [4.10]	12.69 [4.34]	< 0.001
Smoking				< 0.001
Never (*n* (%))	1444 (46.1)	273 (13.5)	1717 (33.3)	
Former (*n* (%))	1317 (42.0)	1327 (65.4)	2644 (51.2)	
Current (*n* (%))	373 (11.9)	428 (21.1)	801 (15.5)	
Years of weekly extreme binge drinking (mean [SD])^b^	3.34 [9.34]	6.95 [12.97]	4.76 [11.05]	< 0.001
Use of other illicit drugs				< 0.001
Yes (*n* (%))	21 (0.7)	564 (27.8)	585 (11.3)	
No (*n* (%))	3113 (99.3)	1463 (72.2)	4576 (88.7)	
Charlson Comorbidity Index score (mean [SD])	0.87 [1.55]	0.82 [1.54]	0.85 [1.55]	0.279
Psychiatric disorders				< 0.001
Yes (*n* (%))	518 (16.5)	556 (27.4)	1074 (20.8)	
No (*n* (%))	2616 (83.5)	1472 (72.6)	4088 (79.2)	

^a^ Analyzed using Welch's *t*‐test.

^b^ Years of weekly extreme binge drinking (≥ 10 units on the same occasion) from the age of 15 to follow‐up.

The association between the predictors (i) cannabis use, (ii) age of initiation of cannabis use, and (iii) years of frequent cannabis use, respectively, and the outcome age‐related cognitive decline from early adulthood to late midlife was analyzed using linear regression. The assumptions for the linear regression model were assessed and fulfilled. All analyses were carried out using SPSS 29.0.0.0.

Initial analyses of interaction were conducted to examine potential interactions between the use of other illicit drugs and all three exposure variables. No significant interactions were found.

Five regression models, including different covariates, were tested in each analysis: An unadjusted model; Model 1: age at follow‐up, retest interval, IQ at conscription, and years of education; Model 2: Model 1 + years of extreme binge drinking, smoking, and use of other illicit drugs; Model 3: Model 1 + psychiatric history and CCI; and Model 4: a fully adjusted model including all mentioned covariates.

## 3 Results

### 3.1 Characteristics of the Study Population

The study population consisted of 5162 men, with a mean cognitive decline of 6.2 IQ points over an average of 44 years. Among the total population, 39.3% had used cannabis at least once. Cannabis users had a slightly higher average IQ at conscription, while the mean IQ difference between cannabis users and nonusers was somewhat larger at the follow‐up. Differences in mean ages and retest interval were also small but significant, while more substantial differences were observed for lifestyle and psychiatric disorders. Cannabis users had a higher proportion of current or former smokers, more years of extreme binge drinking, and a substantially higher proportion with a history of use of other illicit drugs. Finally, a higher proportion of the cannabis users had previous hospital diagnoses with psychiatric disorders.

### 3.2 Cannabis Use and Cognitive Decline

In Table 2, the reference group consisted of nonusers. This group had an unadjusted mean cognitive decline of 6.8 IQ points (SD = 9.5) (see Table 1). Results of both the unadjusted and adjusted models showed significantly less cognitive decline among cannabis users compared to nonusers. In the unadjusted model, cannabis use was associated with 1.5 IQ points less cognitive decline than the decline among nonusers, and in the fully adjusted model, the decline was 1.3 IQ points less among cannabis users compared to nonusers.

**TABLE 2:** Association of cannabis use with IQ changes in unadjusted and adjusted linear regression analyses (*n* = 5162).

	Unadjusted			Model 1^a^			Model 2^b^			Model 3^c^			Model 4^d^		
	*b* [95% CI]^e^	*R* ^2^	*p* value	*b* [95% CI]^e^	*R* ^2^	*p* value	*b* [95% CI]^e^	*R* ^2^	*p* value	*b* [95% CI]^e^	*R* ^2^	*p* value	*b* [95% CI]^e^	*R* ^2^	*p* value
Use of cannabis		0.006			0.191			0.202			0.206			0.213	
Nonusers (*n* = 3134)	Ref.		—	Ref.		—	Ref.		—	Ref.		—	Ref.		—
Users (*n* = 2028)	1.53 [0.99, 2.08]		<0.001	1.37 [0.88, 1.87]		<0.001	1.26 [0.69, 1.83]		<0.001	1.58 [1.09, 2.07]		<0.001	1.28 [0.71, 1.85]		<0.001

^a^ Model 1: Age at follow‐up, retest interval, IQ at conscription, years of education.

^b^ Model 2: Age at follow‐up, retest interval, IQ at conscription, years of education, years of weekly extreme binge drinking, use of other illicit drugs, smoking.

^c^ Model 3: Age at follow‐up, retest interval, IQ at conscription, years of education, psychiatric disorders, CCI.

^d^ Model 4: Fully adjusted model.

^e^ Positive numbers indicate less change in IQ scores from baseline to follow‐up compared to the change observed in the reference group, while negative numbers indicate a larger change in IQ scores compared to the reference group.

### 3.3 Age of Initiation of Cannabis Use and Cognitive Decline

Among cannabis users, 51.1% had their cannabis use initiation before the age of 18, while initiation was between the ages of 18 and 25 for 43.5% and after the age of 25 for 5.4% (data not shown). In Table 3, the reference group consisted of cannabis users who initiated cannabis use after the age of 25. This group experienced an unadjusted mean cognitive decline of 5.8 IQ points (SD = 10.1) (data not shown). Results of both the unadjusted and adjusted models showed a nonsignificant association between the age of initiation of cannabis use and cognitive decline.

**TABLE 3:** Association of age of initiation of cannabis use with IQ changes among cannabis users (*n* = 2028).

	Unadjusted			Model 1^a^			Model 2^b^			Model 3^c^			Model 4^d^		
	*b* [95% CI]^e^	*R* ^2^	*p* value	*b* [95% CI]^e^	*R* ^2^	*p* value	*b* [95% CI]^e^	*R* ^2^	*p* value	*b* [95% CI]^e^	*R* ^2^	*p* value	*b* [95% CI]^e^	*R* ^2^	*p* value
Age of initiation of cannabis use		0.003			0.219			0.226			0.223			0.229	
> 25 years (*n* = 109)	Ref.		—	Ref.		—	Ref.		—	Ref.		—	Ref.		—
18–25 years (*n* = 883)	−0.08 [−2.05, 1.88]		0.933	0.61 [−1.14, 2.36]		0.491	0.52 [−1.22, 2.27]		0.558	0.54 [−1.20, 2.29]		0.542	0.45 [−1.30, 2.19]		0.613
< 18 years (*n* = 1036)	1.14 [−0.81, 3.09]		0.250	1.16 [−0.58, 2.89]		0.191	1.00 [−0.75, 2.75]		0.263	1.16 [−0.57, 2.89]		0.187	0.95 [−0.79, 2.70]		0.285

^a^ Model 1: Age at follow‐up, retest interval, IQ at conscription, years of education.

^b^ Model 2: Age at follow‐up, retest interval, IQ at conscription, years of education, years of weekly extreme binge drinking, use of other illicit drugs, smoking.

^c^ Model 3: Age at follow‐up, retest interval, IQ at conscription, years of education, psychiatric disorders, CCI.

^d^ Model 4: Fully adjusted model.

^e^ Positive numbers indicate less change in IQ scores from baseline to follow‐up compared to the change observed in the reference group, while negative numbers indicate a larger change in IQ scores compared to the reference group.

### 3.4 Years of Frequent Cannabis Use and Cognitive Decline

Information regarding years of frequent cannabis use was only available for LiKO‐15 (*n* = 1114). Among cannabis users, 78.3% had never had a frequent (at least twice a week) use of cannabis, whereas 10.1% had been frequent cannabis users for less than 10 years and 11.7% had been frequent cannabis users for 10 years or more (data not shown). In Table 4, the reference group was the group without frequent use of cannabis. This group experienced an unadjusted mean cognitive decline of 4.5 (SD = 8.9) (data not shown). Results of both the unadjusted and adjusted models showed no significant differences in cognitive decline between men with and without frequent cannabis use.

**TABLE 4:** Association of years of frequent cannabis use (at least twice a week) with IQ changes in a subsample of cannabis users (*n* = 1114).

	Unadjusted			Model 1^a^			Model 2^b^			Model 3^c^			Model 4^d^		
	*b* [95% CI]^e^	*R* ^2^	*p* value	*b* [95% CI]^e^	*R* ^2^	*p* value	*b* [95% CI]^e^	*R* ^2^	*p* value	*b* [95% CI]^e^	*R* ^2^	*p* value	*b* [95% CI]^e^	*R* ^2^	*p* value
Years of frequent cannabis use		0.000			0.202			0.213			0.208			0.217	
No frequent use (*n* = 872)	Ref.		—	Ref.		—	Ref.		—	Ref.		—	Ref.		—
≤ 10 years (*n* = 112)	0.30 [−1.55, 2.15]		0.749	0.63 [−1.04, 2.30]		0.458	0.46 [−1.24, 2.16]		0.598	0.86 [−0.81, 2.53]		0.310	0.59 [−1.11, 2.29]		0.497
> 10 years (*n* = 130)	1.20 [−0.54, 2.93]		0.175	0.49 [−1.07, 2.06]		0.537	0.43 [−1.28, 2.14]		0.620	0.86 [−0.72, 2.44]		0.287	0.59 [−1.12, 2.30]		0.501

^a^ Model 1: Age at follow‐up, retest interval, IQ at conscription, years of education.

^b^ Model 2: Age at follow‐up, retest interval, IQ at conscription, years of education, years of weekly extreme binge drinking, use of other illicit drugs, smoking.

^c^ Model 3: Age at follow‐up, retest interval, IQ at conscription, years of education, psychiatric disorders, CCI.

^d^ Model 4: Fully adjusted model.

^e^ Positive numbers indicate less change in IQ scores from baseline to follow‐up compared to the change observed in the reference group, while negative numbers indicate a larger change in IQ scores compared to the reference group.

## 4 Discussion

### 4.1 Main Findings

In this study of 5162 Danish men, the mean cognitive decline was found to be 6.2 IQ points over an average of 44 years. Notably, cannabis users exhibited statistically significantly less cognitive decline compared to nonusers. In the fully adjusted model, cannabis use was associated with 1.3 IQ points less cognitive decline than the decline observed in the reference group. However, the estimated difference in cognitive decline between cannabis users and nonusers was modest (corresponding to 7% of a standard deviation) and may not hold clinical significance. Among cannabis users, no significant associations with age‐related cognitive decline could be demonstrated for age of initiation of cannabis use. Years of frequent cannabis use were generally associated with no significant difference in cognitive decline when compared with no frequent use.

### 4.2 Comparison With Previous Studies

Few studies with comparable long‐term follow‐up periods exist, and most found no differences in cognitive decline between cannabis users and nonusers. However, the limited studies on this topic employed different methods than those utilized in the present study, making direct comparisons less straightforward. However, our findings do align with results from previous studies indicating no *greater* age‐related cognitive decline associated with cannabis use. In an Australian longitudinal study, 1897 men and women aged 40–46 were followed over 8 years, and it was found that cannabis use was not associated with accelerated cognitive decline in middle‐aged adults (McKetin et al. 2016). Similarly, an American longitudinal study followed 1318 men and women aged 18–64 over 12 years, finding no differences in cognitive decline between heavy users, light users, and nonusers of cannabis (Lyketsos et al. 1999). Finally, in an American longitudinal study of 297 older adults with HIV, occasional cannabis users demonstrated better overall cognitive performance compared to nonusers, although the rates of cognitive decline and everyday function did not vary with the level of cannabis use (Watson et al. 2023).

The observed association of less cognitive decline among cannabis users compared to nonusers in this study may reflect characteristics of cannabis users rather than the direct effects of cannabis itself. For example, cannabis users tended to have higher baseline IQ and education levels, and they tended to smoke more tobacco and consume more alcohol. Hence, it is reasonable to assume that additional unmeasured factors might influence the association, potentially confounding the result. Nevertheless, our findings of less cognitive decline among cannabis users compared to nonusers align with previous in vivo studies indicating that cannabinoids have a positive impact on cognitive function and memory in rats (Marchalant et al. 2008) and mice (Bilkei‐Gorzo et al. 2017; Sarne et al. 2018).

The lack of associations of both age of initiation of cannabis use and years of frequent cannabis use with age‐related cognitive decline among cannabis users does not align with a longitudinal cohort study from New Zealand (Meier et al. 2012). In this study, 1037 men and women were followed from birth (1972/1973) to the age of 38 with a neuropsychological assessment at the age of 13, before the initiation of cannabis use, and again at the age of 38, after the development of a consistent use of cannabis (Meier et al. 2012). Frequent cannabis use was associated with a decline in IQ in various domains, with the largest decline in users with initiation in adolescence (Meier et al. 2012). However, the age period was not comparable with the present study, and the findings might, to a larger degree, reflect the effect of current cannabis use, whereas our study investigates adult life cannabis use with a low proportion of current users. Furthermore, the definition of frequent cannabis use differed from our definition, as frequent cannabis use was defined as 4 days or more per week, whereas in the present study it was defined as a couple of times a week or more. Several studies suggest that the negative effects of cannabis on cognitive functions can be reversed with prolonged abstinence (Schreiner and Dunn 2012; Schulte et al. 2014; Tait, Mackinnon, and Christensen 2011). Adverse effects were not apparent after 3 months of sustained abstinence, even in former heavy users, indicating that frequent cannabis use may not cause irreversible damage (Schulte et al. 2014; Tait, Mackinnon, and Christensen 2011.). In the present study, the majority (92.4%) of cannabis users had not used cannabis in the year leading up to the follow‐up, which could explain the lack of negative effects on cognition due to potential recovery from any cognitive damage caused by previous cannabis use. Moreover, the outcomes might be influenced by a multitude of other variables, particularly given that the average follow‐up duration in the present study spans 44 years.

### 4.3 Methodological Considerations

#### 4.3.1 Strengths

The setup of the DanACo cohort allowed the investigation of the cognitive decline of 5162 men from early adulthood to late midlife using the same intelligence test at baseline and follow‐up. The long retest interval of on average 44 years minimized the practice effect, ensuring more reliable cognitive decline estimations (Grønkjær et al. 2019). The timing of the intelligence assessments was also advantageous, with conscription measurements capturing fluid intelligence close to its peak, enabling a more accurate assessment of cognitive decline. At the follow‐up examination, the age‐related cognitive decline was likely to have started in most men, but the risk of diseases causing pathological cognitive decline would still be relatively low (Grønkjær et al. 2019). In addition, the study's strength lies in the availability of comprehensive data on predictors and covariates obtained from follow‐up questionnaires and the Danish national health registers.

#### 4.3.2 Limitations

While questionnaires and registers allowed for the inclusion of potential confounders, unmeasured or residual confounding might still be present. A potential confounder could be personality differences. However, we found that including measures of personality did not have a significant impact on the association between cannabis use and cognitive decline and thus did not include them in the analyses. Information concerning cannabis use was based on retrospective self‐reports from the follow‐up examination. Thus, recalling detailed information about cannabis use and other factors may have been challenging, particularly for men experiencing greater cognitive decline. However, cannabis users have generally been found to have an accurate recall of both their usage and age of initiation (Shillington et al. 1995). The use of self‐reporting also necessitates considering the risk of social desirability bias. Since the questionnaire addresses sensitive topics like illicit drug use and alcohol consumption, participants may have underreported their usage, potentially introducing bias into the analysis. However, the anonymity of participants was expected to reduce the risk of social desirability bias.

The main limitation of the present study is the low participation rate in the DanACo cohort. Only 14.3% of the invited men participated in the follow‐up examinations, which may affect the generalization of the results and increase the risk of selection bias. It has been shown that the participants of the DanACo cohort had higher intelligence test scores, lower morbidity (CCI), and higher education compared to non‐participants (Grønkjær et al. 2024). It is possible that men with heavy cannabis use, currently as well as previously, were less likely to participate in the follow‐up studies. However, it is not obvious that their willingness to participate would be dependent on their cognitive decline. In addition, the study population consists exclusively of men, and generalization of the results to women may thus not be possible due to the gender‐dependent effects of cannabis use on cognitive functioning (Noorbakhsh et al. 2020; Schnakenberg Martin et al. 2021).

In the present study, the sample size was reduced in the analyses regarding age of initiation of cannabis use and years of frequent cannabis use by including only cannabis users. In addition, the analysis of frequent use was limited to LiKO‐15, and most cannabis users had their cannabis debut before the age of 25, with relatively few using cannabis frequently. Thus, non‐significant findings may result from insufficient statistical power. As the brain of people under 25 years of age is assumed to be more vulnerable due to ongoing development and maturation (Arain et al. 2013; Lubman, Cheetham, and Yücel 2015), early cannabis use may affect cognitive development and complicate assessing age‐related cognitive decline. Furthermore, the present study did not distinguish between cannabis use before and after the conscription board examination, nor frequent use before and after brain maturation. If the men had frequent cannabis use during their cognitive test at conscription, it could have resulted in an artificially low baseline IQ score. In addition, the study could not differentiate between cannabis and other drug use in different age periods, with 27.8% of users having tried other drugs, possibly leading to residual confounding in the analysis of frequent cannabis use.

## 5 Conclusion

In the present study, we aimed to investigate the relationship between cannabis use and age‐related cognitive decline from early adulthood to late midlife. This study contributes to the sparse knowledge on this subject and aligns with most existing studies, suggesting no association between cannabis use and *greater* cognitive decline. More specifically, in the present study, cannabis users experienced slightly less cognitive decline compared to nonusers, and the association remained significant when controlling for potential confounders. Among cannabis users, no significant association was found with cognitive decline for either age of initiation of cannabis use or frequent cannabis use. Further studies are needed to investigate whether these findings reflect that there are no adverse effects on cognitive decline or that the effects of cannabis are temporary and disappear after a prolonged period of time.

## Author Contributions

**Kirstine Maarup Høeg**: conceptualization, methodology, data curation, formal analysis, visualization, writing–original draft, writing–review and editing. **Rasmus Ljungbeck Frodegaard**: conceptualization, methodology, data curation, formal analysis, visualization, writing–original draft, writing–review and editing. **Marie Grønkjær**: writing–review and editing, methodology, data curation. **Merete Osler**: funding acquisition, writing–review and editing, methodology. **Erik Lykke Mortensen**: writing–review and editing, funding acquisition, methodology. **Trine Flensborg‐Madsen**: writing–review and editing, supervision, conceptualization, methodology. **Gunhild Tidemann Okholm**: funding acquisition, writing–review and editing, supervision, data curation, conceptualization.

## Ethics Statement

The LiKO‐15 and DiaKO‐19 studies were submitted for ethics approval by the Committee on Health Research Ethics in the Capital region, but the Committee ruled that according to Danish law (Scientific Ethical Committees Act (in Danish: Komitéloven), article 14, paragraph 2) approval was not required as the studies did not involve collection of biological material.

## Consent

Informed consent was obtained from all participants.

## Conflicts of Interest

The authors have no financial or non‐financial competing interests to declare.

### Peer Review

The peer review history for this article is available at https://publons.com/publon/10.1002/brb3.70136.

## Supporting information




**Table S1**: Frequency of use of euphoriants during different age periods

## Data Availability

The data analyzed in this study are available from the authors, but restrictions apply to the availability of these data. For the current study the data were used under license from the data inspection authorities at University of Copenhagen, and so are not publicly available. However, the data are available from the authors upon reasonable request and with permission from relevant authorities. Queries regarding data access and more information about the cohort can be directed to Gunhild Tidemann Okholm (gunh@sdu.dk or gunhild.tidemann.okholm@regionh.dk).
