# Supplementary material for: Cannabis Use and Age‐Related Changes in Cognitive Function From Early Adulthood to Late Midlife in 5162 Danish Men
Source: Brain Behav. 2024 Nov 7;14(11):e70136. doi: 10.1002/brb3.70136 (PMC11541857; doi:10.1002/brb3.70136)
Supplement: Supplementary file 1 — Table S1: Frequency of use of euphoriants during different age periods [file BRB3-14-e70136-s001.docx]

# Supplementary material

***Table S1:*** *Frequency of use of euphoriants during different age periods.*

|  | Never / Almost never | Less than once a month | Approximately once a month | A couple of times a month | Approximately once a week | A couple of times a week | Every day / Almost every day | Not relevant |
| --- | --- | --- | --- | --- | --- | --- | --- | --- |
| How often have you taken euphoric drugs:  At the age of 14 or under? | 1,054 | 27 | 6 | 7 | 10 | 12 | 8 | 1,380 |
| Over the age of 15-18? | 526 | 204 | 57 | 113 | 86 | 80 | 58 | 1,380 |
| Over the age of 19-25? | 483 | 236 | 84 | 111 | 72 | 84 | 54 | 1,380 |
| Over the age of 26-30? | 799 | 109 | 36 | 55 | 35 | 48 | 42 | 1,380 |
| Over the age of 31-40? | 907 | 73 | 20 | 32 | 22 | 27 | 43 | 1,380 |
| Over the age of 41-50? | 971 | 48 | 18 | 24 | 17 | 17 | 29 | 1,380 |
| Over the age of 51-60? | 1,006 | 46 | 11 | 13 | 13 | 17 | 18 | 1,380 |
| Within the last 12 months? | 1,039 | 26 | 9 | 13 | 5 | 18 | 14 | 1,380 |
| Over the age of 60? | 631 | 11 | 4 | 9 | 5 | 8 | 5 | 1,831 |
